# Tobacco Use during Pregnancy and Its Associated Factors in a Mountain District of Eastern Nepal: A Cross-Sectional Questionnaire Survey

**DOI:** 10.3389/fpubh.2017.00129

**Published:** 2017-06-06

**Authors:** Ramesh Barakoti, Anup Ghimire, Achyut Raj Pandey, Dharani Dhar Baral, Paras K. Pokharel

**Affiliations:** ^1^Public Health Section, Group for Technical Assistance, Lalitpur, Nepal; ^2^Department of School of Public Health and Community Medicine, B.P. Koirala Institute of Health Sciences, Dharan, Nepal; ^3^Research Section, Nepal Health Research Council, Kathmandu, Nepal

**Keywords:** Nepal, pregnancy, prevalence, tobacco, smoking

## Abstract

**Background:**

Tobacco using among women is more prevalent in Nepal as compared to other South-East Asian countries. The effect of its use is seen not only on the pregnant women, but also health of the growing fetus is compromised. Currently, little is known about the tobacco use among women especially during pregnancy in Nepal. This study explored the tobacco use prevalence and its associated factors during pregnancy.

**Materials and methods:**

A cross-sectional study was conducted in Sankhuwasabha, a mountain district of eastern Nepal. Representative sample of 436 women of reproductive age group with infant were selected by stratified simple random sampling. Data were collected by face-to-face interviews of selected participants. Data were analyzed with SPSS version 16.0. Binary logistic regression was used to analyze the relationship among variables.

**Results:**

The study revealed that the prevalence of tobacco use during pregnancy was 17.2%. Only one fifth of the research participants were asked to quit tobacco by health workers during last pregnancy. Multivariable analyses revealed that illiteracy (AOR: 2.31, CI: 1.18–4.52), more than two parity (AOR: 2.45, CI: 1.19–5.07), alcohol use during last pregnancy (AOR: 3.99, CI: 1.65–9.68), and having tobacco user within family (AOR: 2.05, CI: 1.11–3.78) are more likely to use tobacco during pregnancy.

**Conclusion:**

Tobacco use during pregnancy was widely prevalent. Tobacco-focused interventions are required for antenatal women to promote cessation among user and prevent initiation with focus on overcoming problems like illiteracy, high parity, alcohol use, and having other tobacco user family members in family.

## Introduction

Tobacco use is the major public health problem and foremost preventable cause of mortality and morbidity in the world today. Tobacco epidemic is responsible for 5.4 million global deaths annually (1). Tobacco related deaths among women aged 20 years and above may rise from 1.5 million in 2004 to 2.5 million by 2030 and almost 75% of these projected death will be occurring in low- and middle-income countries (2). While overall prevalence of global tobacco use among men is declining slowly, the use of tobacco among women is increasing rapidly. The women from developing countries are at a higher risk (3, 4).

A multi country study has revealed that levels of current smoking were 18.3% in Uruguay, 10.3% in Argentina, 6.1% in Brazil, 3% in Pakistan, and 0.8% each in Ecuador and Guatemala (5). In another study conducted in Canada, the prevalence of smoking during pregnancy was 23% (6). Pooled prevalence of any tobacco use in pregnant women in LMICs was 2.6% (95% CI: 1.8–3.6) with the lowest prevalence in the African region (2.0%, 1.2–2.9) and the highest in the South-East Asian region (5.1%, 1.3–10.9) (7).

One of the previous study had revealed that 14% of women were using tobacco in context of Nepal (8). A hospital based study from eastern Nepal found out that any tobacco use during pregnancy was 19.2% (9). Studies have revealed that tobacco use among women is more prevalent in Nepal as compared to other South-East Asian countries (10).

Using tobacco during pregnancy does not only affect mother, but also the fetus health. Deep vein thrombosis, pulmonary embolism, myocardial infraction, ectopic pregnancy, placenta previa in mother and preterm birth, still birth, stunted gestational development, congenital heart disease in child are associated with tobacco use during pregnancy (11, 12). Despite having knowledge on adverse health effects of tobacco use during pregnancy, it was found that the habit of tobacco consumption does not significantly change before and after getting pregnant (13).

As a co-signatory of ICPD, 1994, Nepal has developed National Reproductive Health Strategy in 1998, which has incorporated awareness raising activities on risk factors including tobacco in integrated reproductive health package (14). House of representative of Nepal ratified FCTC in 2006. The national anti-tobacco communication campaign strategy is in place (15). According to World Health Organization, the first step in treating tobacco use and dependence is to identify tobacco users. Effective identification of tobacco use status opens the door for successful interventions and guides clinicians to identify appropriate interventions based on the tobacco use status of pregnant women and their willingness to quit (16). However, in Nepalese context, data on prevalence of tobacco use in pregnant women are insufficient. In this context, the study attempted to find out tobacco use and its determinants among pregnant women in mountain district of eastern Nepal.

## Materials and Methods

It was a community based, cross-sectional study conducted in Sankhuwasabha district of eastern Nepal among women of reproductive age (15–49 years) having at least one child of less than 1 year.

The sample size was calculated based on the assumption that prevalence of tobacco use during pregnancy was 30% (17). The population living in 33 Village Development Committee (VDC) was considered to represent rural population while population living in the only municipality of the district was considered to represent urban population. Out of 33 VDCs, 4 VDCs were selected randomly from each Illaka using random method. All households of the selected VDC were visited so as to identify pregnant women based on their self reported status. Among 13 wards of the municipality 2 were selected randomly to represent urban representation. Where a single household had more than one pregnant woman, one pregnant women was selected randomly to prevent over representation. Of the total research participants selected, 19.03% were living in urban and municipality areas and 80.96% were living in rural and village areas, which are close to national scenario where the urban population (17%) and rural population (83%).

Face-to-face interview was carried out using a Nepali version of semi-structured questionnaire. Total of 436 participants agreed for the interview out of 440 eligible participants approached.

The study collected information on both forms of tobacco use-smoking and smokeless and tobacco use was defined to include both. Those who can read and write and perform simple mathematical exercise were considered literate based on their self reported ability to read and write. Household refers to a single person living alone or a group of persons, who may or may not be related, usually living in a particular housing unit and sharing meal with common resources. In this way, resident domestic servant was also included as a member in the household. The age of the respondent was left open ended at the time of data collection and recoded afterward in three categories; 15–24, 25–34, and 35–44. Participants were asked about the average monthly income of each working member of the family, which was summed up to determine total family income. Those participants with per capita income less than US$ 1.25 per day were considered poor based on international definition given by World Bank (18). Participants were asked about the religion they follow in four response categories: Hindu, Buddhist, Christian, and other based on most common religions in the country.

Exposure to cigarette smoking during pregnancy was defined as the consumption of a smoking cigarette or bidi or combination of these two at least once during the last pregnancy. It was assessed based on the question, “did you smoke tobacco during last pregnancy?” Those who had smoked were further requested to specify the frequency and type of tobacco (smoking cigarette or bidi or combination of any two) used. Exposure to smokeless tobacco (chewing tobacco, snuff) during pregnancy was defined as the consumption of smokeless tobacco at least once during the last pregnancy. It was assessed based on the question, “did you use smokeless tobacco during last pregnancy?” with response category “yes” and “no.” Drinking during pregnancy is defined as consumption of at least one alcoholic drink during the most recent pregnancy based on their self reported status on question whether they drank alcohol during last pregnancy. Participants who had consumed alcohol during last pregnancy were further asked about the frequency and amount of alcohol use. Pregnant mother who had been consuming tobacco at any point of time during last pregnancy and managed to stop tobacco use and maintain it till the birth was considered as successful quitting of tobacco use.

Status of antenatal check up was assessed by asking question on whether they had visited health facilities during pregnancy. Those who had visited health facility for ANC check up were asked about the frequency of visit in an open ended question, which was later categorized into four visits and less than four visits as per the Nepalese standard on ANC visits. Perinatal complication was determined through separate question on whether they had spontaneous abortions, premature births, stillbirths, placenta previa, placental abruption, and a shorter gestation period with response category yes or no. Positive response to any of these questions was defined as having perinatal complication.

Data collected were checked for completeness and were entered in Microsoft Excel for further cleaning of the data. The Statistical Package for Social Sciences version 16.0 (SPSS Inc., Chicago, IL, USA) was used for analysis.

Chi-square test was used in bivariate analysis for screening of the potential variables for multivariable analysis. Variables that were significant at *p*-value 0.20 were considered for multivariable analyses. Binary logistic regression was applied in multivariable analysis setting level of significance at 5%. Hosmer and Lemeshow test was used to find out fit of the binary regression model. It has been found that this model was fit with χ^2^ (7) = 15.764, *p* = 0.0277.

Ethical approval was taken from the Institutional Review Committee (IRC) and B.P. Koirala Institute of Health Sciences. Written informed consent was taken from each respondent. Participants were explained about the objectives of the study, approximate duration of the interview, potential harm and benefits of participating in the interview, and confidentiality of the information provided. Participants were explained that they would be allowed withdraw at any point of interview without providing any reason to the research team. In case of illiterate research participants, finger impression along with the signature of one witness was considered. Confidentiality and anonymity of the research participants were assured and maintained.

## Results

Of the total 436 research participants, mean age was 25.54 years (SD ±5.95) and mean age at first birth was 20.19 years (SD ±3.23) years. Majority of the research participants (81%) were from rural area and 41.5% had per capita income below poverty line. Almost 77% of the research participants were literate. Likewise most of the research participants (90.4%) were engaged in agriculture whereas only three forth of their husband involved in agriculture. Majority of the research participants (77.1%) were Hindu by religion. Similarly the most common ethnic community was Janajati (78.0%) followed by Brahmin/Chhetri (14.2), and Dalit (7.8%). Almost half of the research participants (49.5%) were living in a nuclear family (Table 1).

**Table 1:** Socio-demographic characteristics of research participants.

Characteristics	Categories	Frequency	Percentage
**Socio-demographic characteristics**			
Age (years)	15–24	231	53.0
	25–34	163	37.4
	35–44	42	9.6

Residence	Rural	353	81.0
	Urban	83	19.0

Education	Illiterate	100	23.0
	Primary	126	28.9
	Secondary	188	43.1
	Higher education	22	5.0

Education status of the husband	Illiterate	34	7.8
	Primary	149	34.2
	Secondary	216	49.5
	Higher education	37	8.5

Occupation	Agriculture	394	90.4
	Business and service	42	9.6

Occupation of the husband	Agriculture	341	78.2
	Labor	50	11.5
	Business and service	45	10.3

Religion	Hindu	336	77.1
	Buddhist	61	14
	Christianity	30	6.9
	Others (Kirat, heavenly path)	9	2.0

Ethnicity	Bhramin/Chhetri	62	14.2
	Janajati	340	78.0
	Dalit	34	7.8

Family type	Nuclear	216	49.5
	Joint	220	50.5

Nearly one third (32.8%) of the research participants were married before age of 18 years and more than two fifth of the research participants (42.2%) had given birth to the first baby before the age of 20 years. Almost two third (66.7%) of the research participants had children ≤2 years (IQR, 1–3). During last pregnancy, 61.5% had at least four antenatal checkup, 18.3% had experienced of perinatal complication(s) in newborn like still birth, low birth weight, small for date, or preterm delivery, and 21.3% were suffered from at least one pregnancy related problem (Table 2).

**Table 2:** Maternal and perinatal history of respondents.

Characteristics	Categories	Frequency	Percentage
Age at marriage	<18 years	143	32.8
	≥18 years	293	67.2

Age at first birth	<20 years	184	42.2
	≥20 years	252	57.8

Number of children	≤2	291	66.7
	3–4	116	26.6
	>4	29	6.7

ANC check up	<4 times	172	39.5
	≥4 times	264	61.5

Perinatal complication on child	No	210	77.8
	Yes	60	22.2

Health problem/s in last 3 months	No	343	78.7
	Yes	93	21.3

Prevalence of ever tobacco user was 17.9% and tobacco in during last pregnancy was 17.2% (95% CI: 13.7–20.7). Nearly one fourth (72.4%) of the users smoked more than two stick of cigarettes per day and more than half (54.5%) of the respondent had five or more snuff of tobacco per day. Twenty percentage of research participants had attempted to quit tobacco use during last pregnancy, which equals to the proportion of participants counseled health worker to quit tobacco. Among participants who attempted quit tobacco, only four were able to quit. Few research participants (3.9%) did cultivate tobacco at household level. Around half of the research participants had at least one member in family who smokes (Table 3).

**Table 3:** Tobacco use by the research participants and family members.

Characteristics	Categories	Frequency	Percentage
Ever use of tobacco	Yes	78	17.9
	No	358	82.1

Household tobacco production	Yes	17	3.9
	No	419	96.1

Tobacco user in Family	Yes	240	55.1
	No	196	44.9

**Tobacco use during last pregnancy**			
Tobacco use	Yes	75	17.2
	No	361	82.8

Stick of cigarette per day	Less than two	8	27.6
	Two or more	21	72.4

Snuff of tobacco per day	Less than five	25	45.5
	Five or more	30	54.5

Attempt to quit tobacco	Yes	15	20.0
	No	60	80.0

Recommended to quit tobacco by health worker	Yes	15	20.0
	No	60	80.0

Success in quitting tobacco	Yes	4	26.7
	No	11	73.3

### Factors Associated with Tobacco Consumption during Pregnancy

After adjusting for other variables, illiterate mothers were twice (AOR = 2.308, 95% CI: 1.179–4.516) more likely to use tobacco during pregnancy compared to literate. Similarly, women having more than two children had higher odds being tobacco user (AOR = 2.454, 95% CI: 1.187–5.072) compared to those having one or two children.

Women having some other members in family who uses tobacco were two times (AOR = 2.049, 95% CI: 1.437–5.041) more likely to use tobacco compared to those who do not have.

Similarly, women who had history of alcohol consumption during last pregnancy were four times more likely (AOR = 3.998, 95% CI: 1.652–9.675) to use tobacco than the women who did not consume alcohol (Table 4).

**Table 4:** Factors associated with tobacco consumption during pregnancy.

Characteristics	Unadjusted OR	95% CI	Adjusted OR	95% CI
**Age (years)**				
Up to 25			Ref.	
More than 25	4.321	2.537–7.360	1.804	0.851–3.820

Educational status*				
Illiterate	4.284	2.528–7.259	2.308	1.179–4.516
Literate			Ref.	

**Educational status of the respondent’s husband**				
Illiterate	1.833	0.818–4.105	0.554	0.213–1.438
Literate			Ref.	

**Occupation**				
Agriculture	0.476	0.231–0.979	0.725	0.207–2.548
Business or service			Ref.	

**Occupation of the respondent’s husband**				
Agriculture	0.720	0.327–1.582	0.465	0.123–1.754
Business or service			Ref.	

**Ethnicity**				
Dalit and Janajati	2.003	1.001–4.128	0.667	0.189–2.357
Bhramin and Chhetri			Ref.	

**Family type**				
Nuclear	0.600	0.362–0.995	0.944	0.493–1.806
Joint			Ref.	

Number of children*				
Up to two			Ref.	
More than two	0.179	0.105–0.305	2.454	1.187–5.072

Alcohol use during last pregnancy*				
Yes	5.174	2.576–10.39	3.998	1.652–9.675
No			Ref.	

Tobacco user in family*				
Yes	2.616	1.507–4.543	2.049	1.111–3.779
No			Ref.	

The sign (*) indicates significant association.

## Discussion

This study revealed that the prevalence of tobacco use during pregnancy was 17.2% (CI: 13.7–20.7), which is comparable the findings from previous studies in Eastern Nepal (19.2%) (9) and in Uruguay (18.3%) (19). The prevalence was higher than reported in Cambodia (13.0%), Argentina (10.3%), Brazil (6.2%), Pakistan (3%), Ecuador (0.8%), and Guatemala (0.8%) (19). These variations across countries could possibly be because of differences in prevailing tobacco control policies of the respective countries, awareness among pregnant women regarding the harmful effects of tobacco use during pregnancy, and the acceptance of tobacco use among women in the community. There are evidences that health care policies significantly affect the likelihood that smokers will receive effective tobacco dependence treatment and successfully stop tobacco use (20). Such differences could have played a role to create differences in overall prevalence of tobacco use among pregnant women across countries.

Evidence suggests that though some women quit when they become pregnant, many other continue to use tobacco throughout the duration of pregnancy (21). In this study, among the total tobacco users, 20% had attempted to quit smoking that equals to proportion of research participants counseled by health workers to quit smoking. Relatively higher proportion of the smokers was asked to quit tobacco in a study conducted in India (46.3% for smokers and 26.7% for users of smokeless tobacco) (22). In one meta analysis that evaluated the effectiveness of health personnel advice on quitting tobacco use including 17 trials of brief advice (as part of a minimal intervention) versus no advice (or usual care) had come out with the finding that advice can significantly increase in quit rates (RR = 1.66, CI: 1.42–1.94) (23). This can be potential area for development of intervention for prevention or quitting tobacco use in context of Nepal as almost four out of every five pregnant women had not received any advice on tobacco cessation. However, insufficient counseling for tobacco cessation among pregnant women can also be because research participants did not reveal their smoking status to health workers or were not screened for their smoking status. In such instances nicotine screening can be beneficial.

Similar to studies done by Nisar et al. in Karachi (Pakistan) (24), illiterate research participants were found to be more likely to use tobacco than the literate. After adjustment of other variables, education level of the research participant was statistically associated with tobacco use in line to findings from some other studies (17, 25–27). Reason behind protective effects of education may be because people tend to avoid harm related tobacco by avoiding its use when they come to know about it through education (8). Education may also increases the likelihood that the people come through informative booklets and health messages relating to harmful effects of tobacco use during pregnancy thereby sensitizing them and encouraging them to quit tobacco. Furthermore, as suggested in one of the previous study, higher prevalence of tobacco use among women with lower education level can be because higher social acceptance of tobacco use among illiterate women (8).

Women who have someone who uses tobacco in family were twice more likely (AOR = 2.049, 95% CI: 1.111–3.779) to use tobacco during pregnancy as compared to those who did. In another study, women whose husbands smoked were twice more likely to smoke (28). The possible explanation would be easy acceptance of tobacco use in family and curiosity that arises about the different aspects of tobacco use when people have someone who consumes tobacco in the family. In this study, women who had more than two children were twice more likely to use tobacco during pregnancy. Similar finding, AOR = 2.54 (CI: 1.82–3.54) was reported in the study done by Ranjit et al. (26). The possible explanation for this relationship might be stress is higher among mother whose numbers of children is greater than national average. According to a recent survey in American mothers, women having three children were more stressful than the women having one or two (19). However, it can be potential area for further research.

Alcohol users at any time of last pregnancy were fourfold more likely to use tobacco. Several studies show that people who drink alcohol often also smoke and vice versa.

Pregnancy has often been referred to as a teachable moment because of mothers’ strong motivation to protect the well being of the fetus or newborn and social pressure to avoid smoking during pregnancy (29). Antenatal visits to the health facility could be opportunity for counseling Nepalese pregnant women for quitting tobacco sensitizing them about its harmful effect in the health of the child and the mother as almost 96% of pregnant women have at least one antenatal visit to health facility (30).

Effective tobacco interventions require coordinated interventions. Just as the health personnel must intervene with his or her patient, so it is must that the health care administrator or public health practitioners foster and support tobacco intervention as an integral element of health care delivery. Health care administrators and/or public health practitioners should ensure that clinicians or other health personnel have the training and support to deliver consistent and effective intervention to tobacco users (20).

Limitation of this study, since tobacco use status was not validated by biomarkers in this study. Retrospective data were collected up to 1 year back, so there is chance of recall bias. Relatively small geographic area, which do hinder to generalize study result national wide.

## Conclusion

Tobacco use during pregnancy was widely prevalent in study district. Furthermore, four variables viz. illiteracy, high parity, alcohol use, and women from tobacco user family member(s) were found to be significantly associated with tobacco use. Thus health care providers and policy makers need to give special attention in those issues and effective implementation of national guideline for effective curving tobacco consumption epidemic during pregnancy.

## Ethics Statement

Ethical approval was taken from the IRC and B.P. Koirala Institute of Health Sciences, which are approved from Nepal Health Research Council. Written informed consent was obtained from the research participants. In case of illiterate research participants, finger impression along with the signature of one witness was considered. Participants were explained about the objectives of the study, approximate duration of the interview, potential harm and benefits of participating in the interview, and confidentiality of the information provided. Participants were explained that they would be allowed withdraw at any point of interview without providing any reason to the research team. In case of illiterate research participants, finger impression along with the signature of one witness was considered. Confidentiality and anonymity of the research participants were assured and maintained.

## Author Contributions

RB: contributed on conception or design of the work; collection, analysis, or interpretation of data, drafting the manuscript, earlier approval, and checking the accuracy or integrity of any part of the work. AG: substantial contributions to the conception of the study and revising critically the manuscript. AP: contributed substantially on analysis and interpretation of data, and revising the manuscript critically for intellectual content. DB: contributed on design of the work; analysis and interpretation of data for the work and revising the manuscript. PP: contributed on conception of the research, interpretation of the data, and revision of the manuscript.

## Conflict of Interest Statement

The authors declare that the research was conducted in the absence of any commercial or financial relationships that could be construed as a potential conflict of interest.
